# Insights Into Vaginal Bacterial Communities and Metabolic Profiles of *Chlamydia trachomatis* Infection: Positioning Between Eubiosis and Dysbiosis

**DOI:** 10.3389/fmicb.2018.00600

**Published:** 2018-03-28

**Authors:** Carola Parolin, Claudio Foschi, Luca Laghi, Chenglin Zhu, Nicoletta Banzola, Valeria Gaspari, Antonietta D’Antuono, Barbara Giordani, Marco Severgnini, Clarissa Consolandi, Melissa Salvo, Roberto Cevenini, Beatrice Vitali, Antonella Marangoni

**Affiliations:** ^1^Department of Pharmacy and Biotechnology, University of Bologna, Bologna, Italy; ^2^Microbiology, Department of Experimental, Diagnostic and Specialty Medicine, University of Bologna, Bologna, Italy; ^3^Centre of Foodomics, Department of Agro-Food Science and Technology, University of Bologna, Bologna, Italy; ^4^Dermatology, Department of Experimental, Diagnostic and Specialty Medicine, University of Bologna, Bologna, Italy; ^5^Institute of Biomedical Technologies – National Research Council, Milan, Italy

**Keywords:** *Chlamydia trachomatis*, eubiosis, bacterial vaginosis, vaginal microbiota, vaginal metabolome

## Abstract

The vaginal microbiota plays a crucial role in maintaining the health and functioning of the female genital tract, preventing the colonization of urogenital pathogens and sexually transmitted infections. In this study, we characterized the vaginal bacterial communities and the metabolome associated to *Chlamydia trachomatis* infection (CT: 20 women), compared to healthy condition (H: 22 women) and bacterial vaginosis (BV: 19 women). A microarray-based tool (VaginArray), implemented with a real-time PCR for *Gardnerella vaginalis*, was used to determine the vaginal bacterial composition, whereas the metabolic profiles were assessed by a proton-based nuclear magnetic resonance (^1^H-NMR) spectroscopy. CT infection was characterized by bacterial and metabolic signatures similar to healthy condition, even though higher amounts of *Lactobacillus iners*, as well as depletion of some amino acids, biogenic amines, and succinate marked CT infection. Moreover, the frequency of *Lactobacillus crispatus* was higher in asymptomatic CT-positive patients than in women with CT-correlated symptoms. We also confirmed the marked differences in the microbiome and metabolome between healthy and BV-affected women. In conclusion, we highlighted microbial and metabolic peculiarities of the vaginal ecosystem in the case of CT infection, even though further studies are needed to understand if the observed alterations precede the infection onset or if the pathogen itself perturbs the vaginal environment.

## Introduction

*Lactobacillus* species play a crucial role in maintaining the health and functioning of the female genital tract, preventing the colonization of urogenital pathogens and sexually transmitted infections (STIs) (Parolin et al., 2015; Nardini et al., 2016; Foschi et al., 2017a; Ñahui Palomino et al., 2017). Their protective role is exerted through the production of antimicrobial compounds, competitive exclusion for adhesion and modulation of the host immune response and membrane functions (Petrova et al., 2015; Calonghi et al., 2017). In particular, lactobacilli benefit the host by producing lactic acid as a fermentation product that lowers the vaginal pH to ∼3.5–4.5 (Ma et al., 2012).

The depletion of lactobacilli, together with the increase of different species of anaerobes, can result in the switch from a normal vaginal microbiota to a dysbiosis known as bacterial vaginosis (BV) (Lambert et al., 2013). Molecular studies based on 16S rRNA gene have shown that lactobacilli are dominant in the majority of healthy women (H), while in case of BV, the vaginal niche is characterized by higher abundances of different bacteria, including *Gardnerella vaginalis*, *Atopobium*, *Prevotella*, *Mycoplasma hominis, Mobiluncus*, and *Veillonella* (Vitali et al., 2007; Dols et al., 2016).

The marked changes in the bacterial communities during BV are associated with profound perturbations in the composition of vaginal metabolites (Yeoman et al., 2013; Laghi et al., 2014; McMillan et al., 2015; Vitali et al., 2015).

On the other hand, little information is available about the vaginal bacterial and metabolic signatures in *Chlamydia trachomatis* (CT) cervical infection. CT, an obligate intracellular pathogen, represents the causative agent of the most common bacterial STI worldwide. A significant proportion of urogenital CT infections in women is asymptomatic and, if left untreated, can lead to several complications and sequelae (Haggerty et al., 2010).

Several observational studies have demonstrated that BV is an independent risk factor for STIs acquisition, including CT (Wiesenfeld et al., 2003; Allsworth and Peipert, 2011). Besides these epidemiological reports, only few evidence exists on the vaginal bacterial communities of women with an ongoing CT infection (Filardo et al., 2017; van der Veer et al., 2017). Moreover, no data are available about the metabolic fingerprints of CT infection.

The aim of this study was to characterize the vaginal microbiome and the metabolome associated with CT infection, in comparison with eubiosis (H) and a model of dysbiosis (BV). A microarray-based tool (VaginArray), targeting the most representative vaginal bacteria (Cruciani et al., 2015), implemented with a real-time PCR for *G. vaginalis*, was used to determine the composition of the vaginal bacterial communities. A proton-based nuclear magnetic resonance (^1^H-NMR) spectroscopy was used to assess the metabolic profiles.

## Materials and Methods

### Study Group and Sample Collection

From January to July 2016, all the pre-menopausal non-pregnant Caucasian women attending the STI Outpatients Clinic of Sant’Orsola-Malpighi Hospital in Bologna (Italy) and meeting one of the following criteria were enrolled: presence of urogenital symptoms and/or presence of risk factors for CT infection (age < 25 years, new or multiple sexual partners, unsafe intercourses).

Exclusion criteria comprised the use of any antibiotics or vaginal medications in the past month, the use of estro-progestinic products, the presence of chronic diseases, HIV-seropositivity and a state of obesity of II° grade. Moreover, patients with vulvo-vaginal candidiasis, trichomoniasis, aerobic vaginitis, *Neisseria gonorrhoeae* (GC) and *Mycoplasma genitalium* infections were excluded.

For all patients, demographic data, behavioral characteristics and information about urogenital symptoms were recorded. After a clinical examination, two vaginal swabs were collected from each woman. The first one (E-swab, Copan, Brescia, Italy) was used for microbiology tests and BV assessment. Nucleic acid amplification techniques (NAATs) were used for CT, GC, *Trichomonas vaginalis* and *M. genitalium* detection by Versant CT/GC DNA 1.0 Assay (Siemens Healthineers, Tarrytown, NY, United States), Aptima *Trichomonas vaginalis* and Aptima *M. genitalium* assays (Panther system, Hologic, Marlborough, MA, United States), respectively. Microscopic examination and culture were performed for candidiasis and aerobic vaginitis diagnosis. BV assessment was based on the presence of three of the four Amsel criteria, together with the presence of a Nugent score > 3 (Vitali et al., 2015).

Eligible women were allocated in one of the following groups: H (absence of symptoms and negative microbiological tests), BV (positivity for Amsel criteria together with a Nugent score > 3) or CT (detection of CT DNA by NAATs).

The second vaginal swab was collected with a sterile cotton bud, re-suspended in 1 ml of sterile saline and stored at -80°C until use. Frozen vaginal swabs were thawed, vortexed for 1 min and removed from the liquid. The liquid was centrifuged at 10000 × *g* for 15 min, in order to separate cell pellets from cell-free supernatants.

This study was carried out in accordance with the recommendations of the Ethics committee of the Sant’Orsola-Malpighi Hospital (Bologna) with written informed consent from all subjects. All subjects gave written informed consent in accordance with the Declaration of Helsinki. The protocol was approved by the Ethics committee of the Sant’Orsola-Malpighi Hospital (7/2016/U/Tess).

### Analysis of Vaginal Communities by VaginArray

Bacterial communities were determined by means of VaginArray, a phylogenetic DNA-microarray able to detect the most representative species of the human vaginal microbiota, under both eubiosis and dysbiosis (Cruciani et al., 2015). Genomic DNA was isolated from vaginal cell pellets by using a DNeasy Blood and Tissue Kit (Qiagen, Hilden, Germany) and 16S rRNA gene was amplified with universal primers 27F and 1492R. PCR products were purified by using a High Pure PCR Clean up Micro kit (Roche, Mannheim, Germany). Universal arrays (UAs) were prepared on the surface of phenylen-diisothiocyanate activated chitosan glass slides (Cruciani et al., 2015). Ligase detection reactions (LDRs) and hybridization of the products on the UAs were performed as previously described (Parolin et al., 2017). Briefly, LDRs were carried out with 48 ng of purified PCR products, added with 250 fmol of a synthetic template for normalization purposes. Arrays were scanned by using a ScanArray 5000 scanner (Perkin Elmer Life Sciences, Boston, MA, United States) at 10 μm resolution and the fluorescence intensity (IF) was quantified by ScanArray Express 3.0 software. IFs were subjected to normalization. Significantly present spots were determined using a one-tailed *t*-test (*P* < 0.05) comparing, for each bacteria-associated probe, the distribution of IFs along all replicates with the distribution of IFs of negative controls.

### Real-Time PCR for *Gardnerella vaginalis*

A single-plex quantitative real-time PCR (qPCR) targeting the 16S rRNA gene of *G. vaginalis* (GV) was performed on vaginal genomic DNA, as previously described (Cox et al., 2015). A standard curve was prepared using serial dilutions of quantified genomic GV DNA (ATCC^®^ 49145D-5, ATCC, Manassas, VA, United States). Results were expressed as GV DNA copies/reaction.

### ^1^H-NMR Metabolomic Analysis

Metabolomic analysis was performed starting from 700 μl of the cell-free supernatants of the vaginal swabs, according to Vitali et al. (2015). The signals originating from large molecules were suppressed by a CPMG filter of 400 echoes, generated by 180° pulses of 24 μs separated by 400 μs (Ventrella et al., 2016). The signals were assigned by comparing their multiplicity and chemical shift with Chenomx software data bank (ver 8.1 Chenomx, Inc., Edmonton, AB, Canada).

### Statistical Analysis

Data were analyzed with Prism 5.02 version for Windows (GraphPad Software, San Diego, CA, United States). Differences in clinical and demographic parameters were tested by Chi-square test or ANOVA test. Similarities among microbial and metabolic profiles of samples were investigated by R computational language^1^. Clustering and heatmaps were generated by heatmap.2 function of the gplots R-package. VaginArray and qPCR data, as well as metabolites concentrations, were centered and scaled to unity variance. VaginArray and qPCR data were subjected to principal components analysis (PCA), while metabolomics data were analyzed by robust PCA (RPCA) (Hubert et al., 2005). In both cases, correlation between original variables and PC was assessed by Pearson correlation. Differences in microbial contents or metabolites concentrations and PCA/RPCA data were analyzed by Wilcoxon’s signed rank test. Differences in microbial contents among H, BV and CT groups were analyzed by Kruskal–Wallis test, followed by Dunn’s *post hoc* test adjusted for multiple comparison. A *P*-value < 0.05 was considered as statistically significant, unless otherwise stated.

The relationship between microbiota and metabolome profiles was studied by means of co-inertia analysis (Dolédec and Chessel, 1994), while the strength of such correlation was estimated by RV-coefficient (Robert and Escoufier, 1976).

## Results

### Study Group

During the study period a total of 170 women were enrolled and, out of these, 61 patients were considered eligible. Specifically, 22 were considered healthy (36.1%), 19 received a diagnosis of BV (31.1%), and 20 were positive for CT infection (32.8%). The remaining 109 patients were excluded from the study, because of the presence of other clinical conditions, as trichomoniasis, gonorrhea, vulvo-vaginal candidiasis, aerobic vaginitis, *M. genitalium* infection, or mixed infections.

No cases of clinical evident BV with a contemporary CT infection were found. Clinical, behavioral and demographic information of the study groups are reported in details in **Table 1**.

**Table 1:** Demographic, behavioral, and clinic characteristics of the women enrolled for the study.

	Healthy (*N* = 22)	BV (*N* = 19)	CT (*N* = 20)	*P-*value
**Enrolment criteria**	No symptoms and negative for microbiologic tests	Positive for 3/4 Amsel criteria and Nugent score > 3	Vaginal detection of CT DNA by NAAT	

**Mean age ± *SD* (years)**	26.2 ± 6.2	28.5 ± 6.9	24.3 ± 3.4	0.08

**Mean BMI (kg/m^2^) ± *SD***	23.8 ± 2.4	22.9 ± 2.4	23.6 ± 1.8	0.4

**Sexual orientation**				
Heterosexual	22/22 (100%)	19/19 (100%)	20/20 (100%)	-
**Previous STIs**				
Syphilis	3/22 (13.6%)	1/19 (5.3%)	3/20 (15%)	0.58
Urogenital CT infection	4/22 (18.2%)	3/19 (15.8%)	3/20 (15%)	0.95
Genital HPV warts	1/22 (4.5%)	0/19 (0%)	1/20 (5%)	0.60
**Amsel criteria**				
Creamy gray discharge	0/22 (0%)	16/19 (84.2%)	0/20 (0%)	<0.0001
Mean vaginal pH values	4.1 ± 0.3	4.8 ± 0.4	4.2 ± 0.4	<0.0001
Positive Whiff test^a^	6/22 (27.3%)	10/19 (52.6%)	4/20 (20%)	0.07
Clue cells present	1/22 (4.5%)	18/19 (94.7%)	3/20 (15%)	<0.0001
**Nugent score**				
0–3	17/22 (77.3%)	0/19 (0%)	13/20 (65%)	<0.0001
4–6	5/22 (22.7%)	8/19 (42.1%)	7/20 (35%)	0.40
7–10	0/22 (0%)	11/19 (57.9%)	0/20 (0%)	<0.0001
**Presence of urogenital symptoms**	0/22 (0%)	19/19 (100%)	9/20 (45%)	<0.0001


*^*a*^The Whiff test is considered positive when, by adding a drop of 10% KOH to a microscopic slide containing the vaginal secretions, a characteristic ‘fishy’ odor is present.*

While all the patients with BV complained about various genital symptoms, more than half (11/20) of CT patients was completely asymptomatic. Complained symptoms of CT-infected women were the presence of vaginal discharge (6/9) and dyspareunia (5/9) and, less frequently, dysuria (2/9) and abnormal bleeding (2/9).

Most of the women in the H and CT groups were characterized by a Nugent score 0–3 (77.3 and 65%, respectively), opposite to BV subjects showing a Nugent score > 7 in 57.9% of cases.

### Vaginal Microbiome

The composition of the vaginal microbiome was analyzed by the VaginArray, implemented with a qPCR for the quantitative detection of GV (Supplementary Tables S1, S2).

**FIGURE 1:**
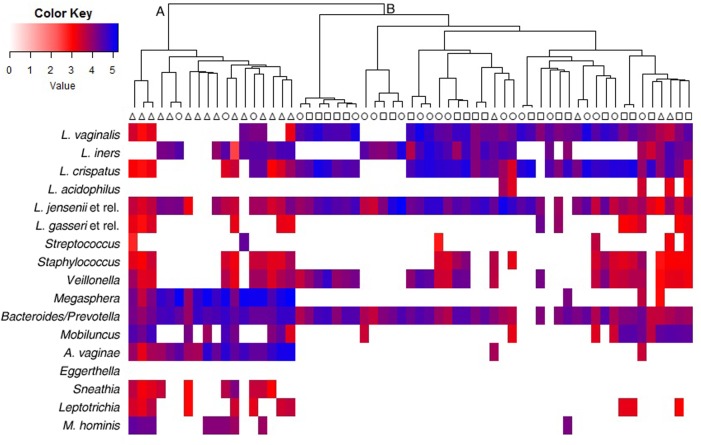
Heatmap and hierarchical clustering of the VaginArray data. Squares, triangles, and circles represent H, BV, and CT women, respectively. The mean values of the logarithm of the fluorescence intensity for each bacterial group are plotted in a color scale. Maximum distances and Ward’s clustering method were used to create the dendrogram.

IF signals obtained from VaginArray were represented as a heatmap in **Figure 1**. Hierarchical clustering identified two main groups of women: cluster A and cluster B. Indeed cluster A included most BV patients (15/19) and only 3 out of 20 CT women, whereas all H subjects fell in cluster B along with most of CT women. BV subjects included in cluster B (4/19) were characterized by the positivity of Amsel criteria and Nugent score in the range 4–6, whereas CT women included in cluster A (3/20) showed Nugent scores 4–6, but the contemporary negativity for Amsel criteria. Significant differences in clustering were observed for BV subjects with respect to both H and CT women (*P* < 0.0001).

**FIGURE 2:**
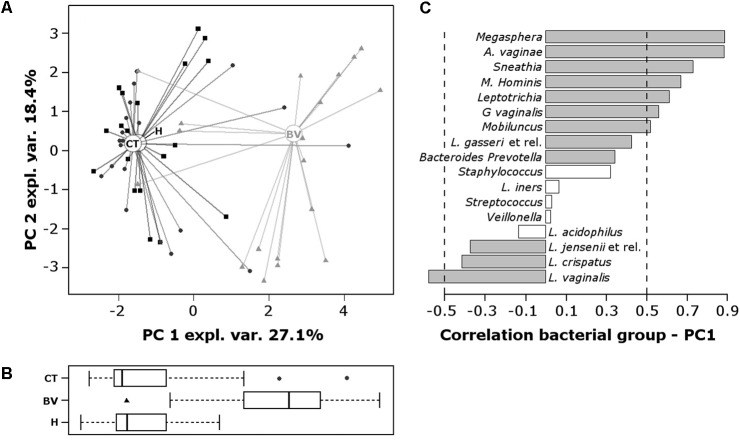
Principal components analysis (PCA) of the vaginal bacterial communities of H, BV, and CT subjects. PCA was performed on centered and scaled data. **(A)** Score-plot of PCA. The squares, triangles, and circles represent H, BV, and CT women, respectively. **(B)** Box plot representing the distribution of PC1 values of the women groups. **(C)** Pearson correlation coefficients between bacterial groups concentration and PC1 values. To ease the visual interpretation of the values, the –0.5 and 0.5 are remarked by dashed lines and significant correlations (*P* < 0.01) are depicted with filled bars.

Data obtained from both the VaginArray and GV qPCR were subjected to a scaled and centered PCA to investigate the correlation between bacterial signatures and women groups (**Figure 2**). In the score-plot (**Figure 2A**), most of CT subjects fell close to H samples, whereas BV subjects were clearly separated from H controls. This feature was better visualized evaluating the distribution of the groups along PC1, which describes the higher contribution to variance (**Figure 2B**): H and CT groups showed similar PC1 median values (*P* = 0.9699), significantly distinct from the BV group (*P* < 0.0001). H group was the most homogeneous one (*SD*: 0.96), indicating that all H subjects bore a similar microbiome, whereas CT and BV groups showed a high degree of variability among the samples (*SD*: 1.71 and 1.95, respectively).

H and CT subjects harbored high amounts of *Lactobacillus* species, particularly *L. vaginalis*, *L. crispatus*, and *L. jensenii*; contrariwise, BV group was characterized by high contents of *Megasphaera*, *A. vaginae*, *Sneathia*, *M. hominis*, *Leptotrichia*, *G. vaginalis*, and *Mobiluncus* (**Figure 2C**).

**FIGURE 3:**
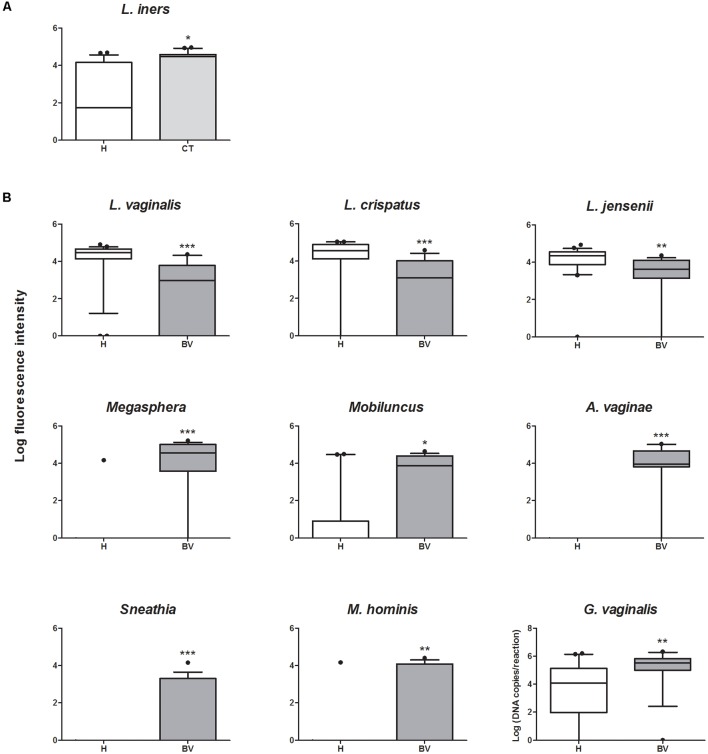
Box plot representing the quantification of bacterial genera/species that significantly vary between groups. **(A)** Quantification of bacteria showing significant variations between CT and H subjects. **(B)** Quantification of bacteria showing significant variations between BV and H subjects. ^∗^*P* < 0.05; ^∗∗^*P* < 0.01; ^∗∗∗^*P* < 0.001.

To better decipher the peculiarities of each group, we focused on the bacterial targets whose contents were differentially detected among the three conditions, and we sought for differences in the comparisons between CT and H women, as well as between BV and H (**Figure 3**). Compared to H group, CT subjects were characterized by significantly higher amounts of *Lactobacillus iners* (*P* = 0.0337) (**Figure 3A**). Concerning the comparison BV vs. H (**Figure 3B**), several significant differences were highlighted for *Lactobacillus* species and anaerobes. In particular, BV women were depleted of *L. vaginalis* (*P* = 0.0003), *L. crispatus* (*P* = 0.0006), and *L. jensenii* (*P* = 0.005), but bore higher amounts of *Megasphaera* (*P* < 0.0001), *Mobiluncus* (*P* = 0.0358), *A. vaginae* (*P* < 0.0001), *Sneathia* (*P* = 0.0009), *M. hominis* (*P* = 0.0085), and *G. vaginalis* (*P* = 0.0078).

### Vaginal Metabolome

Metabolomic analysis quantitatively detected 54 molecules, mainly belonging to the groups of SCFAs, organic acids, amino acids, and biogenic amines (Supplementary Table S3). Differences were searched in the metabolic profiles of CT and BV women, compared to H controls. Our data highlighted differences in the concentration of 10 molecules between CT and H, and in the concentration of 40 molecules between BV and H (Supplementary Table S3).

**FIGURE 4:**
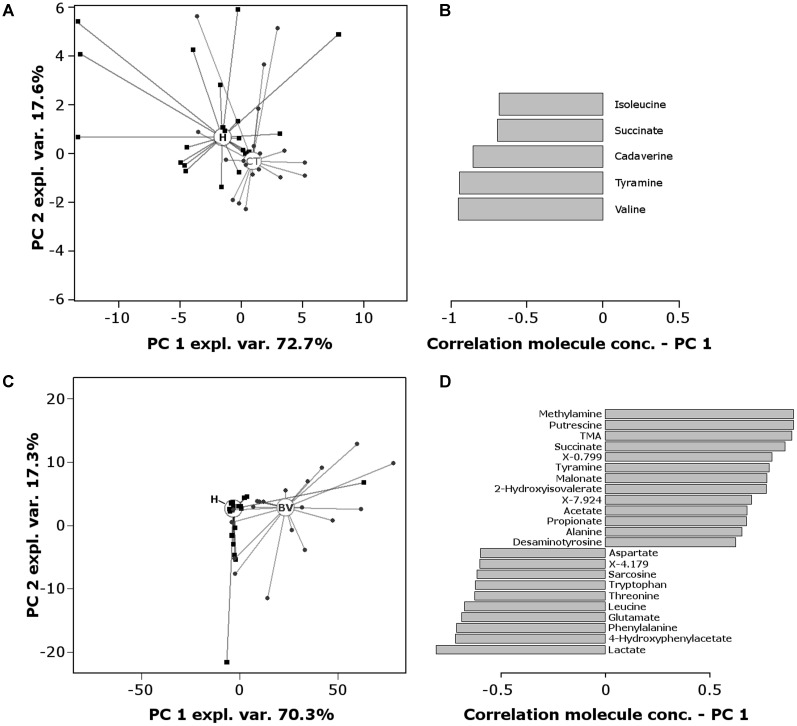
RPCA model built on the centered and scaled concentrations of the metabolites showing significant differences between groups. **(A)** Score-plot of RPCA built on the 10 metabolites that varies between CT and H women. Circles and squares represent CT and H subjects, respectively; empty circles represent the median PC score for each group. **(B)** Pearson correlation coefficients between molecules concentration and PC1 values (comparison CT vs. H). **(C)** Score-plot of RPCA built on the 40 metabolites that varies between BV and H women. Triangles and squares represent BV and H subjects, respectively; empty circles represent the median PC score for each group. **(D)** Pearson correlation coefficients between molecules concentration and PC1 values (comparison BV vs. H). Only molecules with a correlation higher than 0.5 or lower than –0.5 are represented. Correlations were statistically significant for all the molecules (*P* < 0.01).

To obtain overviews of the differences among groups, RPCA models were calculated on the basis of the molecules statistically different between the groups (**Figure 4**). **Figures 4A,B** describe the comparison CT vs. H: in the score-plot (**Figure 4A**), H women showed lower median values along PC1 than CT women (*P* = 0.0068); depletion of valine, isoleucine, tyramine, cadaverine, and succinate seems to be fingerprints of CT infection, as visualized in the correlation plot along PC1 (**Figure 4B**).

**Figures 4C,D** report the comparison BV vs. H: the score-plot (**Figure 4C**) shows a clear separation of the two metabolomes along PC1 (*P* < 0.0001). The correlation plot along PC1 (**Figure 4D**) highlights the molecules that mostly contributed to this separation: (i) biogenic amines (methylamine, putrescine, TMA, tyramine, desaminotyrosine), organic acids (succinate, malonate, 2-hydroxyisovalerate, and SCFAs) and alanine were more concentrated in BV samples; (ii) lactate, 4-hydroxyphenylacetate, diverse amino acids (phenylalanine, glutamate, leucine, threonine, tryptophan, aspartate) and amino acid derivatives (sarcosine), characterized H subjects.

In order to gain an insight on the correlation between the bacterial communities and metabolic profiles, we constructed a co-inertia model (Supplementary Figure S1). The key feature of such model is the RV coefficient, which measures the similitude of the samples distribution across the two parameters (microbiota and metabolome, Supplementary Figures S1A,B). Such coefficient was found, in 0–1 scale, equal to 0.95, thus showing that bacterial and metabolic profiles were tightly intertwined. In particular, lactate and 4-hydroxyphenylacetate appeared strongly related to the presence of lactobacilli, with particular reference to *L. vaginalis*. On the other side, organic acids and biogenic amines appeared strongly related to *Megasphaera* and *A. vaginae*.

### Symptomatic and Asymptomatic *Chlamydia* Infections

We searched for correlations between the vaginal microbiome and metabolome composition and the presence of CT-related clinical signs. A significant difference was found for *L. crispatus* frequency of occurrence: all CT-asymptomatic women harbored *L. crispatus*, whereas this species was retrieved only in half of CT-symptomatic group (five out of nine, *P* = 0.026). No significant difference in the amount of *L. crispatus* between symptomatic and asymptomatic women was found.

Regarding the metabolome, 4-aminobutyrate showed significant different concentrations in asymptomatic (2.08 × 10^-3^± 1.21 × 10^-2^) and symptomatic CT women (1.01 × 10^-2^± 2.72 × 10^-2^) (*P* = 0.027).

## Discussion

While BV has been thoroughly studied in terms of microbial composition and metabolic profiles, less information is available on the vaginal ecosystem during *C. trachomatis* infections.

The relevance of a deep comprehension of the features of the vaginal niche in CT-positive women lies in several aspects: (i) understanding the reasons why some individuals clear the infection and others develop chronic infections (Aiyar et al., 2014); (ii) elucidating the different clinical/immunological pictures of CT infections (Mirmonsef et al., 2011; Petrova et al., 2015); (iii) opening the perspective of new therapeutic approaches based on probiotic lactobacilli to facilitate CT clearance (Ziklo et al., 2016). Recent studies have already highlighted the potential of vaginal lactobacilli to counteract *C. trachomatis* infectious process, mainly through the inactivation of bacterial elementary bodies by lactic acid (Gong et al., 2014; Nardini et al., 2016).

In this study, we investigated the vaginal microbiome and the metabolome in three different clinical conditions: healthy, BV and CT infection. In particular, to explore the bacterial communities and metabolic profiles of the vaginal niche, we integrated a phylogenetic characterization (DNA-microarray/qPCR) with a metabolomic approach (^1^H-NMR).

Considering that many factors can perturb the inhabitants of the vaginal microbiota, such as the ethnicity, the recent use of antibiotics or the intake of estrogenic products (Larsen and Monif, 2001; Ravel et al., 2011), as a strength of our work, we excluded from the study all the women with conditions potentially leading to biases in the results.

Moreover, to the best of our knowledge, this paper applies for the first time ^1^H-NMR-based metabolomics on vaginal swabs, instead of vaginal fluids (Laghi et al., 2014; Vitali et al., 2015). Collection of vaginal swabs is less time-consuming and improves patient compliance, allowing to envisage new perspectives for wide metabolomic studies.

We confirmed the high diversity between the vaginal bacterial communities and metabolic profiles of H and BV women (Yeoman et al., 2013; Vitali et al., 2015; Dols et al., 2016). Despite almost all the women harbored lactobacilli, *L. vaginalis*, *L. crispatus*, and *L. jensenii* mainly characterized the microbiome of healthy women. Contrariwise, BV-affected women showed a minor *Lactobacillus* colonization, in conjunction with higher amounts of strict anaerobes, especially *Megasphaera, Mobiluncus, A. vaginae, Sneathia, M. hominis*, and *G. vaginalis*. Moreover, in accordance with previous investigations (Yeoman et al., 2013; Laghi et al., 2014; Vitali et al., 2015), BV women were characterized by higher levels of biogenic amines, organic acids (especially SCFAs) and alanine, while higher levels of lactate, 4-hydroxyphenylacetate, and diverse aminoacids were associated to the healthy status.

We found that the vaginal microbiome of CT-positive women resembled those of healthy subjects, being characterized by a high colonization of lactobacilli and relatively low occurrences of BV-related bacteria. Notably, we detected a higher colonization of *L. iners* in the vaginal microbiome of CT women compared to H controls, in agreement with recent reports (van der Veer et al., 2017; van Houdt et al., 2017). This finding supports the hypothesis that *L. iners* is a transitional species, colonizing after perturbations of the vaginal environment (Macklaim et al., 2011), including STIs (Srinivasan et al., 2012; Borgdorff et al., 2014). Conversely, *L. crispatus* resulted the hallmark of a healthy vaginal status. This species has been indicated as one of the most active against several pathogens, including CT (Parolin et al., 2015; Nardini et al., 2016; Foschi et al., 2017b), suggesting its potential in the development of new probiotic formulations as therapeutic agents (Vitali et al., 2016).

Interestingly, asymptomatic CT-positive patients harbored *L. crispatus* more frequently than women with symptomatic infection. In this context, it has been reported that *L. crispatus* is able to reduce pro-inflammatory cytokines production in CT-infected cells (Rizzo et al., 2015), pointing out the ability of this species to modulate the pathogen-related immune response.

We are aware of the limitations of this microarray-based technique, in contrast to unbiased approaches (e.g., next-generation sequencing), in particular the partial description of the microbiota composition limited to the selected targets which does not take into account potential alterations in minor components of the microbiota, and the impossibility of making absolute quantification. However, VaginArray allows a fast, reliable, time- and cost-effective determination of the most representative bacterial groups that compose the vaginal ecosystem in both eubiosis and dysbiosis, and can give a first picture of the alterations occurring within the bacterial communities in BV and CT infection.

Concerning the vaginal metabolic profiles, CT samples showed significant reductions in some amino acids and biogenic amines with respect to H women, suggesting a correlation between *C. trachomatis* infection and the metabolism of nitrogen compounds. This result can be interpreted in two directions: (i) *C. trachomatis* may consume nitrogen molecules as preferred nutritional sources or (ii) *C. trachomatis* may impact on the nitrogen metabolism of the infected host cells (Saka et al., 2011; Omsland et al., 2012).

## Conclusion

Although *Chlamydia* infection is not associated with drastic changes in the composition and metabolic activity of the vaginal ecosystem, we observed some microbial and metabolic peculiarities, i.e., abundance of *L. iners*, and shifts in nitrogen metabolism. Cross sectional study design and the relatively small sample size are limitations. Further investigations with a prospective longitudinal study design would afford understanding whether these alterations precede the infection onset or if the pathogen itself perturbs the vaginal environment.

## Author Contributions

BV, AM, and RC conceived and designed the study. NB, VG, and AD’A recruited volunteers and collected the samples. CP, CF, LL, CZ, BG, and MS performed the experiments. CP, CF, LL, MS, and CC analyzed the data. MS, CC, BV, and AM contributed reagents, materials and analysis tools. CP, CF, AM, and BV wrote the paper. All authors read, reviewed, and approved the final manuscript.

## Conflict of Interest Statement

The authors declare that the research was conducted in the absence of any commercial or financial relationships that could be construed as a potential conflict of interest.

## Footnotes

1 www.r-project.org
