# Supplementary material for: Insights Into Vaginal Bacterial Communities and Metabolic Profiles of Chlamydia trachomatis Infection: Positioning Between Eubiosis and Dysbiosis
Source: Front Microbiol. 2018 Mar 28;9:600. doi: 10.3389/fmicb.2018.00600 (PMC5883401; doi:10.3389/fmicb.2018.00600)
Supplement: Supplementary file 1 [file Data_Sheet_1.DOCX]

**Supplementary materials**

**Table S1.** Raw data of the VaginArray analysis. Results are expressed as mean values of fluorescence intensity (n=4 spots for each bacterial target). H: healthy, BV: BV-affected, CT: CT-positive subjects. -: non significant spot, determined by using a one-tailed t-test (*P* = 0.05) comparing, for each Zip Code, the distribution of IFs along all replicates with the distribution of IFs of negative controls.

| ***L. vaginalis*** | | | ***L. iners*** | | | ***L. crispatus*** | | | ***L. acidophilus*** | | | ***L. jensenii* et rel.** | | |
| --- | --- | --- | --- | --- | --- | --- | --- | --- | --- | --- | --- | --- | --- | --- |
| **H** | **BV** | **CT** | **H** | **BV** | **CT** | **H** | **BV** | **CT** | **H** | **BV** | **CT** | **H** | **BV** | **CT** |
| 80907.1 | 1616.5 | - | - | 40100.6 | 91174.9 | 107064.9 | 7938.2 | - | - | - | - | 31123.9 | 16752.6 | 149420.7 |
| 56746.2 | 15071.3 | - | 48265.8 | 29943.6 | 31878.8 | 90564.2 | 25804.8 | - | - | - | - | 25007.5 | 7351.5 | 16868.1 |
| 20676.5 | - | 64040.3 | 46073.5 | 17968.0 | - | 24773.9 | - | 68067.9 | - | - | - | 58250.0 | 14665.8 | 28277.0 |
| 61968.0 | 23688.3 | 28480.1 | - | - | 19500.6 | 61843.9 | 15813.3 | 35112.5 | - | - | - | 25304.4 | 12662.7 | - |
| 49747.0 | - | 115502.1 | - | 9645.5 | 39024.1 | 31558.3 | - | 73275.7 | - | - | - | 12029.0 | 9254.0 | 26295.3 |
| 14219.3 | - | 91454.7 | - | - | - | 13182.2 | - | 30025.3 | - | - | - | 7467.5 | - | 44750.0 |
| 11645.3 | 20884.3 | 28242.1 | - | 23936.1 | 54685.9 | - | 20310.5 | 65488.7 | - | - | - | 12602.6 | 22846.0 | 64059.6 |
| 13320.1 | - | - | 13921.8 | 15808.5 | 29389.4 | 12529.7 | - | - | - | - | - | 43481.7 | 17469.2 | 4165.5 |
| 36391.3 | - | - | - | 39491.7 | 13923.1 | 24604.9 | 231- | - | - | - | - | 32335.4 | 4308.1 | 3134.9 |
| 44982.5 | 3925.4 | 23098.8 | 5119.6 | 39790.4 | 34394.6 | 37515.0 | 37901.1 | 61652.4 | - | 1671.9 | - | 19086.4 | 9262.7 | 14953.6 |
| 39906.1 | 1945.8 | 61057.6 | - | - | 85268.0 | 74757.2 | 1590.2 | 86471.2 | - | - | - | 46043.6 | 4080.6 | 61181.7 |
| - | - | 97733.1 | 18009.8 | - | - | - | 3487.8 | 102414.6 | - | - | - | 85484.2 | 3273.9 | 27305.6 |
| - | - | 16882.6 | 11890.0 | 22194.0 | 6753.0 | - | 1128.0 | 9055.2 | - | - | 2999.5 | 13382.2 | 2631.5 | 7590.5 |
| 50842.4 | - | 30772.4 | 7911.3 | - | - | 57238.9 | - | 55303.9 | - | - | - | 32976.5 | 1392.9 | 3506.9 |
| 35451.0 | 6093.6 | 46898.6 | - | 8336.8 | - | 34134.3 | 10167.1 | 80495.9 | - | - | - | 26819.6 | 1144.8 | 3721.7 |
| 19028.4 | 20204.7 | 21282.7 | 11214.0 | 25895.5 | 31370.3 | 56846.1 | - | 2241.7 | - | - | 2064.2 | - | - | 58897.8 |
| 28222.6 | - | 12820.6 | - | - | 43359.6 | 104720.6 | - | 7960.7 | - | - | 7496.3 | 4678.9 | - | 118848.0 |
| 30778.9 | 2839.5 | 15071.3 | 16154.1 | - | 29943.6 | 2150.0 | 1239.5 | 25804.8 | 1443.4 | - | - | 7489.0 | 3955.6 | 7162.1 |
| 15471.5 | 931.1 | 29891.0 | - | - | - | 78921.4 | 865.0 | 20827.8 | - | - | - | 8244.6 | 2199.3 | 8627.7 |
| 12228.9 |  | - | 3106.1 |  | 30993.5 | 62464.2 |  | 2209.8 | - |  | - | 2038.0 |  | 4618.5 |
| 39636.3 |  |  | - |  |  | 107951.6 |  |  | - |  |  | 44332.3 |  |  |
| 13650.0 |  |  | 20238.4 |  |  | 17399.8 |  |  | - |  |  | 2470.8 |  |  |

| ***L. gasseri* et rel.** | | | ***Streptococcus*** | | | ***Staphylococcus*** | | | ***Veillonella*** | | | ***Megasphera*** | | |
| --- | --- | --- | --- | --- | --- | --- | --- | --- | --- | --- | --- | --- | --- | --- |
| **H** | **BV** | **CT** | **H** | **BV** | **CT** | **H** | **BV** | **CT** | **H** | **BV** | **CT** | **H** | **BV** | **CT** |
| - | 1678.1 | - | - | - | - | - | 6229.3 | - | 22990.4 | 9147.8 | - | - | 161692.0 | - |
| - | - | - | - | - | - | 13378.8 | 3531.9 | - | 20152.0 | 7472.1 | - | - | 119109.1 | 138494.8 |
| - | - | - | - | - | - | - | - | - | - | - | - | - | 40836.6 | - |
| - | - | - | - | - | - | 7437.5 | - | - | 15114.9 | - | - | - | - | - |
| - | - | - | - | - | - | - | - | - | 9021.9 | - | 32188.6 | - | 52267.4 | - |
| 8794.1 | - | - | - | - | - | - | - | - | - | - | 16797.0 | - | 59980.1 | - |
| 19618.1 | - | - | - | - | 720.4 | - | 4748.0 | 2731.7 | 16404.3 | 4239.0 | 3199.1 | - | - | - |
| - | - | - | - | - | - | - | - | - | - | - | - | - | 62970.4 | - |
| - | 1776.7 | - | - | - | - | - | 2058.8 | - | 9496.6 | 5784.0 | - | - | 100227.4 | - |
| - | - | - | - | 1414.8 | - | - | 1202.1 | 2336.4 | 10440.2 | 1757.2 | 2952.9 | - | - | - |
| - | 2359.4 | - | - | - | - | - | 3192.9 | - | 57517.4 | 2247.3 | 25634.9 | - | 15299.8 | - |
| - | 1738.5 | - | - | - | - | - | 1481.9 | - | - | 1716.3 | - | - | 15296.5 | - |
| - | - | 2784.9 | - | - | - | - | 1947.8 | 8266.7 | - | 2311.8 | 5590.3 | - | 36562.7 | 4116.4 |
| - | - | - | - | - | 4093.2 | 6950.7 | - | 2587.1 | 21947.8 | - | 3899.9 | - | 8376.6 | - |
| - | - | - | - | - | - | - | 795.5 | 2645.4 | 12990.3 | 978.5 | 2926.4 | - | 945.9 | - |
| - | - | - | - | 23374.6 | - | - | - | 1743.1 | - | - | - | 14618.8 | 126458.0 | - |
| 1490.9 | - | - | - | - | - | 3041.4 | - | - | 5544.8 | - | - | - | 131490.3 | - |
| 1683.9 | 1726.3 | - | 1406.3 | 535.8 | - | 1316.8 | 1914.4 | 3463.0 | 1489.0 | 8008.7 | 7472.1 | - | 18131.3 | 119109.1 |
| 1171.7 | 886.6 | - | - | - | - | 1981.9 | 1211.3 | - | 3648.3 | 2062.1 | 3537.1 | - | 3755.5 | - |
| - |  | - | - |  | - | - |  | 2755.6 | - |  | 5540.9 | - |  | 122140.1 |
| - |  |  | - |  |  | - |  |  | - |  |  | - |  |  |
| 1226.6 |  |  | - |  |  | 1238.5 |  |  | 1918.3 |  |  | - |  |  |

| ***Bacteroides/Prevotella*** | | | ***Mobilincus*** | | | ***A. vaginae*** | | | ***Eggerthella*** | | | ***Sneathia*** | | |
| --- | --- | --- | --- | --- | --- | --- | --- | --- | --- | --- | --- | --- | --- | --- |
| **H** | **BV** | **CT** | **H** | **BV** | **CT** | **H** | **BV** | **CT** | **H** | **BV** | **CT** | **H** | **BV** | **CT** |
| 41427.8 | 53920.3 | 35305.5 | - | 2075.8 | - | - | 107363.0 | - | - | - | - | - | - | - |
| 31117.5 | 22374.2 | 25500.4 | - | - | - | - | 25235.4 | 28876.6 | - | - | - | - | 3536.5 | - |
| 25281.4 | 46433.2 | - | - | - | - | - | 16568.8 | - | - | - | - | - | - | - |
| 29146.2 | 15274.8 | - | - | - | - | - | - | - | - | - | - | - | - | - |
| 12232.6 | 39180.4 | 33241.6 | - | - | - | - | 22031.7 | - | - | - | - | - | - | - |
| 12464.8 | 53760.6 | 33784.6 | - | - | - | - | 6684.8 | - | - | - | - | - | - | - |
| 13646.2 | 11848.1 | 8189.0 | - | - | - | - | 6568.5 | - | - | - | - | - | - | - |
| 15324.1 | 40786.6 | 6187.6 | - | - | 3384.3 | - | 7468.5 | - | - | - | - | - | 3487.4 | - |
| 11904.7 | 54113.2 | 3809.6 | - | 24799.1 | - | - | 101939.0 | - | - | - | - | - | - | - |
| 20198.1 | 5930.9 | 5187.1 | - | 29948.4 | - | - | - | - | - | - | - | - | - | - |
| 49084.5 | 18920.0 | 25947.8 | - | 44148.4 | - | - | 6544.7 | - | - | - | - | - | 1999.9 | - |
| 18089.9 | 27894.8 | 24838.7 | - | 17080.1 | - | - | 26150.2 | - | - | - | - | - | 14134.1 | - |
| 13229.5 | 23044.6 | 21149.1 | - | 13138.7 | 19708.6 | - | 53736.4 | 3291.4 | - | - | - | - | 1099.2 | - |
| 30940.9 | 8574.9 | 5624.8 | - | 15787.3 | 2113.5 | - | 7728.7 | - | - | - | - | - | 1017.4 | - |
| 34428.7 | 3919.2 | 6434.9 | - | 24985.1 | 2893.7 | - | - | - | - | - | - | - | - | - |
| 23926.0 | 44184.4 | 4772.2 | - | - | 1752.8 | - | 46523.4 | - | - | - | - | - | - | - |
| 14525.9 | 33437.5 | 6832.9 | 30711.2 | 7505.3 | - | - | 70060.4 | - | - | - | - | - | - | - |
| 7355.5 | 16801.2 | 22787.6 | 25233.1 | 33600.6 | - | - | 8805.8 | 25235.4 | - | - | - | - | 4389.9 | 3538.0 |
| 13050.7 | 9367.7 | 5500.1 | 29433.3 | 18649.6 | - | - | 1803.9 | - | - | - | - | - | 1408.5 | - |
| 3740.9 |  | 55609.7 | 4141.3 |  | 40936.6 | - |  | 70758.8 | - |  | - | - |  | 4714.2 |
| - |  |  | - |  |  | - |  |  | - |  |  | - |  |  |
| 8925.3 |  |  | 27796.6 |  |  | - |  |  | - |  |  | - |  |  |

| ***Leptotrichia*** | | | ***M. hominis*** | | |
| --- | --- | --- | --- | --- | --- |
| **H** | **BV** | **CT** | **H** | **BV** | **CT** |
| - | 3848.1 | - | - | - | - |
| - | - | - | - | 8001.1 | - |
| - | - | - | - | - | - |
| - | - | - | - | - | - |
| - | - | - | - | 12092.0 | - |
| - | - | - | - | - | - |
| - | - | - | - | - | - |
| - | - | - | - | - | - |
| - | 2914.6 | - | - | - | - |
| - | - | - | - | - | - |
| - | 4477.8 | - | - | 19976.3 | - |
| - | 2865.1 | - | - | 7999.0 | - |
| - | - | - | - | - | - |
| - | 1038.9 | - | - | - | - |
| - | - | - | - | - | - |
| - | - | - | 14618.8 | - | - |
| 1754.6 | - | - | - | 11922.0 | - |
| - | 2650.3 | 1817.6 | - | 25011.0 | - |
| 1847.8 | 1770.0 | - | - | 20003.9 | - |
| - |  | - | - |  | 11989.0 |
| - |  |  | - |  |  |
| 1149.6 |  |  | - |  |  |

**Table S2.** Raw data of the real-time quantitative PCR, targeting the 16S rRNA gene of *Gardnerella vaginalis* (GV). Results are expressed as GV DNA copies/reaction. H: healthy, BV: BV-affected, CT: CT-positive subjects

| **H** | **BV** | **CT** |
| --- | --- | --- |
| 25200 | 358300 | 370 |
| 0 | 1580000 | 427000 |
| 1380000 | 63000 | 70000 |
| 0 | 413790 | 9500 |
| 15800 | 100000 | 92300 |
| 1570000 | 316000 | 2000 |
| 79400 | 331730 | 280 |
| 63000 | 200000 | 500 |
| 3 | 100000 | 18100 |
| 25200 | 0 | 14000 |
| 630 | 77000 | 36700 |
| 448 | 321400 | 21400 |
| 25 | 1870000 | 20100 |
| 1600 | 574800 | 0 |
| 964100 | 267 | 0 |
| 1360000 | 2110000 | 89 |
| 416 | 1160000 | 590 |
| 614000 | 689400 | 135545 |
| 15800 | 127400 | 433 |
| 0 |  | 1110000 |
| 2300 |  |  |
| 8700 |  |  |

**Table S3.** Concentration (mmol/l) of metabolites determined by ^1^H-NMR. Results are expressed as mean ± standard deviation. H: healthy, BV: BV-affected, CT: CT-positive subjects. Arrows indicate significant variations (*P* < 0.05) in metabolite concentration (↑ increase, ↓ decrease) in BV and CT women compared to healthy control.

|  | **H** | **BV** | **CT** | **CT vs H** | **BV vs H** |
| --- | --- | --- | --- | --- | --- |
| **Amines** |  |  |  |  |  |
| Tyramine | 5.47x10^-02^ ± 1.87x10^-02^ | 6.33x10^-02^ ± 6.65x10^-02^ | 4.22x10^-02^ ± 1.47x10^-02^ | ↓ | ↑ |
| TMA | 1.38x10^-03^ ± 3.92x10^-03^ | 6.71x10^-03^ ± 7.43x10^-03^ | 7.57x10^-04^ ± 1.57x10^-03^ |  | ↑ |
| TMA-Nox | 4.25x10^-03^ ± 7.55x10^-04^ | 4.48x10^-03^ ± 1.18x10^-03^ | 4.82x10^-03^ ± 1.31x10^-03^ |  |  |
| Putrescine | 3.44x10^-02^ ± 7.11x10^-02^ | 1.09x10^-01^ ± 1.17x10^-01^ | 2.23x10^-02^ ± 3.12x10^-02^ |  | ↑ |
| Methylamine | 5.27x10^-04^ ± 1.72x10^-03^ | 4.32x10^-03^ ± 5.20x10^-03^ | 5.44x10^-04^ ± 1.53x10^-03^ |  | ↑ |
| Glutamine | 2.16x10^-02^ ± 9.80x10^-03^ | 1.60x10^-02^ ± 1.21x10^-02^ | 1.85x10^-02^ ± 6.60x10^-03^ |  |  |
| DMA | 1.00x10^-03^ ± 3.72x10^-04^ | 7.56x10^-04^ ± 2.66x10^-04^ | 8.17x10^-04^ ± 3.81x10^-04^ | ↓ |  |
| Ethanolamine | 1.47x10^-02^ ± 3.28x10^-03^ | 1.69x10^-02^ ± 7.08x10^-03^ | 1.49x10^-02^ ± 3.11x10^-03^ |  |  |
| Cadaverine | 5.68x10^-02^ ± 5.22x10^-02^ | 6.36x10^-02^ ± 6.14x10^-02^ | 3.85x10^-02^ ± 1.14x10^-02^ | ↓ |  |
| **Organic acids** |  |  |  |  |  |
| Succinate | 1.22x10^-01^ ± 1.76x10^-01^ | 2.48x10^-01^ ± 2.73x10^-01^ | 6.07x10^-02^ ± 8.67x10^-02^ | ↓ | ↑ |
| Pyruvate | 1.91x10^-02^ ± 1.43x10^-02^ | 6.32x10^-02^ ± 4.82x10^-02^ | 3.05x10^-02^ ± 3.00x10^-02^ |  | ↑ |
| Malonate | 1.74x10^-03^ ± 7.75x10^-04^ | 5.65x10^-03^ ± 3.91x10^-03^ | 2.16x10^-03^ ± 2.94x10^-03^ |  | ↑ |
| Lactate | 30.30 ± 9.22x10^-01^ | 12.50 ± 1-0 | 30.80 ± 9.00x10^-01^ |  | ↓ |
| 4-Hydroxyphenylacetate | 1.77x10^-02^ ± 6.21x10^-03^ | 5.97x10^-03^ ± 6.56x10^-03^ | 1.51x10^-02^ ± 6.41x10^-03^ |  | ↓ |
| 2-Hydroxyisovalerate | 2.12x10^-03^ ± 8.32x10^-04^ | 6.16x10^-03^ ± 3.51x10^-03^ | 2.57x10^-03^ ± 1.47x10^-03^ |  | ↑ |
| Formate | 6.58x10^-02^ ± 3.10x10^-02^ | 1.65x10^-01^ ± 1.17x10^-01^ | 1.07x10^-01^ ± 9.70x10^-02^ |  | ↑ |
| Benzoate | 1.85x10^-03^ ± 2.82x10^-03^ | 2.92x10^-03^ ± 4.31x10^-03^ | 2.36x10^-03^ ± 3.21x10^-03^ |  |  |
| Acetate | 2.48x10^-01^ ± 1.94x10^-01^ | 9.86x10^-01^ ± 6.11x10^-01^ | 3.38x10^-01^ ± 4.94x10^-01^ |  | ↑ |
| Propionate | 1.65x10^-02^ ± 3.37x10^-02^ | 5.14x10^-02^ ± 6.26x10^-02^ | 1.78x10^-02^ ± 2.74x10^-02^ |  | ↑ |
| Isovalerate | 3.17x10^-02^ ± 1.09x10^-02^ | 2.65x10^-02^ ± 1.29x10^-02^ | 3.45x10^-02^ ± 9.62x10^-03^ |  |  |
| Butyrate | 9.15x10^-02^ ± 1.37x10^-01^ | 1.87x10^-01^ ± 2.94x10^-01^ | 9.43x10^-02^ ± 7.05x10^-02^ |  |  |
| **Aminoacids** |  |  |  |  |  |
| Valine | 4.34x10^-02^ ± 1.65x10^-02^ | 3.24x10^-02^ ± 2.63x10^-02^ | 3.37x10^-02^ ± 1.16x10^-02^ | ↓ | ↓ |
| Tryptophan | 4.89x10^-03^ ± 1.60x10^-03^ | 1.85x10^-03^ ± 1.97x10^-03^ | 3.84x10^-03^ ± 1.94x10^-03^ |  | ↓ |
| Threonine | 4.38x10^-02^ ± 1.80x10^-02^ | 2.20x10^-02^ ± 2.16x10^-02^ | 4.47x10^-02^ ± 1.01x10^-02^ |  | ↓ |
| Taurine | 1.31x10^-01^ ± 2.60x10^-02^ | 1.28x10^-01^ ± 3.38x10^-02^ | 1.49x10^-01^ ± 5.06x10^-02^ |  |  |
| Proline | 3.03x10^-03^ ± 1.48x10^-03^ | 8.53x10^-03^ ± 6.91x10^-03^ | 3.26x10^-03^ ± 2.61x10^-03^ |  | ↑ |
| Phenylalanine | 3.15x10^-02^ ± 8.81x10^-03^ | 1.29x10^-02^ ± 1.13x10^-02^ | 2.86x10^-02^ ± 1.18x10^-02^ |  | ↓ |
| Leucine | 1.27x10^-01^ ± 3.47x10^-02^ | 5.18x10^-02^ ± 3.09x10^-02^ | 1.06x10^-01^ ± 4.41x10^-02^ |  | ↓ |
| Isoleucine | 3.34x10^-02^ ± 8.25x10^-03^ | 1.50x10^-02^ ± 1.24x10^-02^ | 2.45x10^-02^ ± 1.11x10^-02^ | ↓ | ↓ |
| Glutamate | 8.37x10^-02^ ± 3.82x10^-02^ | 4.94x10^-02^ ± 3.65x10^-02^ | 9.57x10^-02^ ± 2.88x10^-02^ |  | ↓ |
| Glycine | 1.14x10^-01^ ± 1.98x10^-01^ | 4.46x10^-02^ ± 2.95x10^-02^ | 6.34x10^-02^ ± 1.57x10^-02^ | ↓ | ↓ |
| Aspartate | 1.84x10^-02^ ± 6.67x10^-03^ | 9.04x10^-03^ ± 7.45x10^-03^ | 1.47x10^-02^ ± 7.40x10^-03^ |  | ↓ |
| Alanine | 5.97x10^-02^ ± 1.74x10^-02^ | 7.54x10^-02^ ± 2.24x10^-02^ | 5.67x10^-02^ ± 1.98x10^-02^ |  | ↑ |
| **Alcohols** |  |  |  |  |  |
| Methanol | 3.94x10^-03^ ± 1.59x10^-03^ | 4.65x10^-03^ ± 1.99x10^-03^ | 5.54x10^-03^ ± 3.61x10^-03^ |  |  |
| Isopropanol | 3.63x10^-03^ ± 1.73x10^-03^ | 5.41x10^-03^ ± 2.78x10^-03^ | 3.83x10^-03^ ± 2.06x10^-03^ |  | ↑ |
| Ethanol | 3.69x10^-02^ ± 3.29x10^-02^ | 5.74x10^-02^ ± 5.38x10^-02^ | 3.35x10^-02^ ± 2.47x10^-02^ |  |  |
| **Sugars** |  |  |  |  |  |
| Maltose | 2.38x10^-01^ ± 7.79x10^-01^ | 6.62x10^-02^ ± 6.37x10^-02^ | 1.05x10^-01^ ± 1.09x10^-01^ |  |  |
| Glucose | 7.29x10^-02^ ± 2.87x10^-01^ | 1.66x10^-02^ ± 1.01x10^-02^ | 1.55x10^-02^ ± 1.16x10^-02^ |  | ↓ |
| **Others** |  |  |  |  |  |
| Uracil | 1.56x10^-03^ ± 3.65x10^-03^ | 1.25x10^-03^ ± 4.04x10^-03^ | 9.64x10^-04^ ± 2.44x10^-03^ |  |  |
| sn-Glycero-3-phosphocholine | 9.81x10^-03^ ± 3.49x10^-03^ | 7.42x10^-03^ ± 6.91x10^-03^ | 1.07x10^-02^ ± 3.32x10^-03^ |  | ↓ |
| Sarcosine | 1.27x10^-02^ ± 5.44x10^-03^ | 4.10x10^-03^ ± 3.88x10^-03^ | 8.42x10^-03^ ± 4.27x10^-03^ | ↓ | ↓ |
| O-Acetylcholine | 4.80x10^-03^ ± 1.78x10^-03^ | 8.11x10^-03^ ± 5.14x10^-03^ | 5.69x10^-03^ ± 3.39x10^-03^ |  | ↑ |
| π-Methylhistidine | 9.95x10^-03^ ± 4.56x10^-03^ | 5.44x10^-03^ ± 4.08x10^-03^ | 1.15x10^-02^ ± 5.22x10^-03^ |  | ↓ |
| 3-methyl-2-oxovalerate | 1.16x10^-04^ ± 4.94x10^-04^ | 6.40x10^-04^ ± 9.89x10^-04^ | 2.68x10^-04^ ± 1.56x10^-03^ |  | ↑ |
| Hypoxanthine | 3.98x10^-03^ ± 1.73x10^-03^ | 2.42x10^-03^ ± 3.61x10^-03^ | 4.61x10^-03^ ± 2.87x10^-03^ |  | ↓ |
| Hippurate | 1.01x10^-03^ ± 2.72x10^-03^ | 5.36x10^-04^ ± 1.04x10^-03^ | 7.82x10^-04^ ± 5.40x10^-03^ |  |  |
| 2-Heptanone | 2.69x10^-03^ ± 1.85x10^-03^ | 2.95x10^-03^ ± 2.82x10^-03^ | 3.00x10^-03^ ± 2.01x10^-03^ |  |  |
| 1,3-dihydroxyacetone | 2.77x10^-03^ ± 2.93x10^-03^ | 6.00x10^-04^ ± 4.97x10^-04^ | 2.57x10^-03^ ± 3.40x10^-03^ |  | ↓ |
| Desaminotyrosine | 9.10x10^-03^ ± 1.44x10^-02^ | 1.45x10^-02^ ± 1.38x10^-02^ | 4.39x10^-03^ ± 2.87x10^-03^ |  | ↑ |
| Creatinine | 1.50x10^-02^ ± 1.35x10^-02^ | 9.37x10^-03^ ± 7.66x10^-03^ | 1.01x10^-02^ ± 7.72x10^-03^ | ↓ |  |
| 5-Aminopentanoate | 4.04x10^-02^ ± 2.72x10^-02^ | 6.86x10^-02^ ± 2.80x10^-02^ | 3.60x10^-02^ ± 4.17x10^-02^ |  | ↑ |
| 4-Aminobutyrate | 2.84x10^-02^ ± 5.28x10^-02^ | 4.71x10^-03^ ± 4.33x10^-03^ | 5.87x10^-03^ ± 1.36x10^-02^ | ↓ |  |
| Adenine | 1.82x10^-02^ ± 7.21x10^-03^ | 5.98x10^-03^ ± 8.53x10^-03^ | 1.89x10^-02^ ± 8.76x10^-03^ |  | ↓ |
| **Unassigned signals** |  |  |  |  |  |
| X-8.251 | 1.31x10^-03^ ± 7.99x10^-04^ | 1.11x10^-03^ ± 1.46x10^-03^ | 1.58x10^-03^ ± 9.09x10^-04^ |  |  |
| X-8.231 | 9.03x10^-04^ ± 5.79x10^-04^ | 3.12x10^-04^ ± 5.88x10^-04^ | 1.43x10^-03^ ± 1.39x10^-03^ |  | ↓ |
| X-7.924 | 2.54x10^-03^ ± 4.54x10^-03^ | 5.12x10^-03^ ± 5.35x10^-03^ | 2.82x10^-03^ ± 3.50x10^-03^ |  | ↑ |
| X-7.697 | 3.84x10^-03^ ± 6.65x10^-03^ | 4.76x10^-03^ ± 8.88x10^-03^ | 8.57x10^-03^ ± 1.39x10^-02^ |  |  |
| X-7.569 | 1.81x10^-03^ ± 3.58x10^-03^ | 2.61x10^-03^ ± 5.22x10^-03^ | 4.32x10^-03^ ± 8.36x10^-03^ |  |  |
| X-4.179 | 1.46x10^-02^ ± 9.12x10^-03^ | 4.44x10^-03^ ± 4.10x10^-03^ | 1.53x10^-02^ ± 5.95x10^-03^ |  | ↓ |
| X-2.816 | 2.35x10^-03^ ± 1.05x10^-02^ | 3.67x10^-03^ ± 9.66x10^-03^ | 3.07x10^-04^ ± 1.18x10^-03^ |  |  |
| X-1.654 | 1.81x10^-02^ ± 1.09x10^-02^ | 2.02x10^-02^ ± 1.64x10^-02^ | 1.47x10^-02^ ± 4.19x10^-03^ |  |  |
| X-1.129 | 2.41x10^-02^ ± 9.83x10^-03^ | 2.70x10^-02^ ± 1.17x10^-02^ | 2.23x10^-02^ ± 8.62x10^-03^ |  |  |
| X-0.911 | 4.91x10^-02^ ± 5.54x10^-02^ | 5.96x10^-02^ ± 8.30x10^-02^ | 3.69x10^-02^ ± 2.^01^x10^-02^ |  |  |
| X-0.799 | 8.84x10^-05^ ± 4.52x10^-04^ | 1.06x10^-03^ ± 1.20x10^-03^ | 1.09x10^-04^ ± 2.06x10^-04^ |  | ↑ |

TMA=trimethylamine; DMA=dimethylamine

**Figure S1.** Co-inertia model built on the centred and scaled concentrations of microbiota and metabolome data. **A)** scoreplot calculated on the microbiota. Thick black, dark-gray and light-gray lines link each subject from H, CT and BV groups with the median of the corresponding group. Thin lines show where each sample would fall, if represented by means of the metabolome profile. **B)** scoreplot calculated on the metabolome. Thick black, dark-gray and light-gray lines link each subject from H, CT and BV groups with the median of the corresponding group. Thin lines show where each sample would fall, if represented by means of the microbiota profile. **C-D)** loading-plots of the microorganisms and molecules concentrations.
